# Individualized recovery of gut microbial strains post antibiotics

**DOI:** 10.1038/s41522-019-0103-8

**Published:** 2019-10-11

**Authors:** Hyunmin Koo, Joseph A. Hakim, David K. Crossman, Ranjit Kumar, Elliot J. Lefkowitz, Casey D. Morrow

**Affiliations:** 1 Department of Genetics and Heflin Center for Genomic Science, University of Alabama at Birmingham, Birmingham, AL 35294 USA; 2 Department of Biology, University of Alabama at Birmingham, Birmingham, AL 35294 USA; 3 Biomedical Informatics, Center for Clinical and Translational Sciences, University of Alabama at Birmingham, Birmingham, AL 35294 USA; 4 Department of Microbiology, University of Alabama at Birmingham, Birmingham, AL 35294 USA; 5 Department of Cell, Developmental and Integrative Biology, University of Alabama at Birmingham, Birmingham, AL 35294 USA

**Keywords:** Microbiome, Metagenomics

## Abstract

To further understand the impact of antibiotics on the gastrointestinal tract microbial community, the intra-individual recovery pattern of specific microbial strains was determined using metagenomic sequencing coupled with strain-tracking analyses. In a study where 18 individuals were administered a single antibiotic (cefprozil), new microbial genomic variants (herein strains) were transiently detected in 15 individuals, while in a second study that used a cocktail of three antibiotics (meropenem, gentamicin, and vancomycin), all 12 participants had either permanent or transient strain changes. The presence of distinct microbial genomic variants indicates a pattern of strain recovery that is intra-individual specific following disruption of the human gastrointestinal tract with antibiotics.

## Introduction

Numerous studies have shown the profound impact that antibiotics have on the composition of the gastrointestinal (GI) tract microbial communities.^1–3^ The consensus from these studies is that antibiotics cause a disruption of the microbial composition that can have a long-term impact on the community structure. As a consequence of this disruption, the normal function of the commensal communities is compromised.^2,4^

In the new era of culture-independent analysis, microbial genomic variants (i.e. strains) have been identified in the human microbiome using next-generation sequencing.^5,6^ In a previous study we developed Window-based single-nucleotide variant (SNV) similarity (WSS) to assess the strain relatedness of multiple microbes in two separate samples.^7^ For the given microbes, a pairwise comparison was used to determine the overall genome-wide SNV similarity. Using paired samples from the Human Microbiome Project (HMP) data set, we established a WSS cut-off value for each microbial strain, which can differentiate a related sample pair from a non-related sample pair.^7^

## Results and discussion

In the current study, we have utilized WSS strain-tracking techniques to investigate the impact of antibiotics on the stability and emergence of new strains following perturbation by antibiotics (Supplementary Data 1). The first data set from Raymond et al.^8^ collected fecal samples from 18 individuals at three different time points: pre-treatment (Day 0), end of antibiotic (cefprozil) treatment (Day 7), and 3 months post-treatment (Day 90). Six additional individuals who did not receive antibiotic treatment were added from Raymond et al. to this study as controls. The second data set from Palleja et al.^9^ collected fecal samples from 12 individuals at five different time points: pre-treatment (Day 0), immediately after antibiotics (meropenem, gentamicin, and vancomycin) treatment (Day 4), and three post-treatment time points (Day 8, 42, and 180). From the second data set, only four different time points (Day 0, 8, 42, and 180) were selected and used for further analyses. After the coverage-based filtering process, a total of 30 and 37 species were detected from the Raymond et al., and the Palleja et al. data sets, respectively (Supplementary Data 1).

**Fig. 1:**
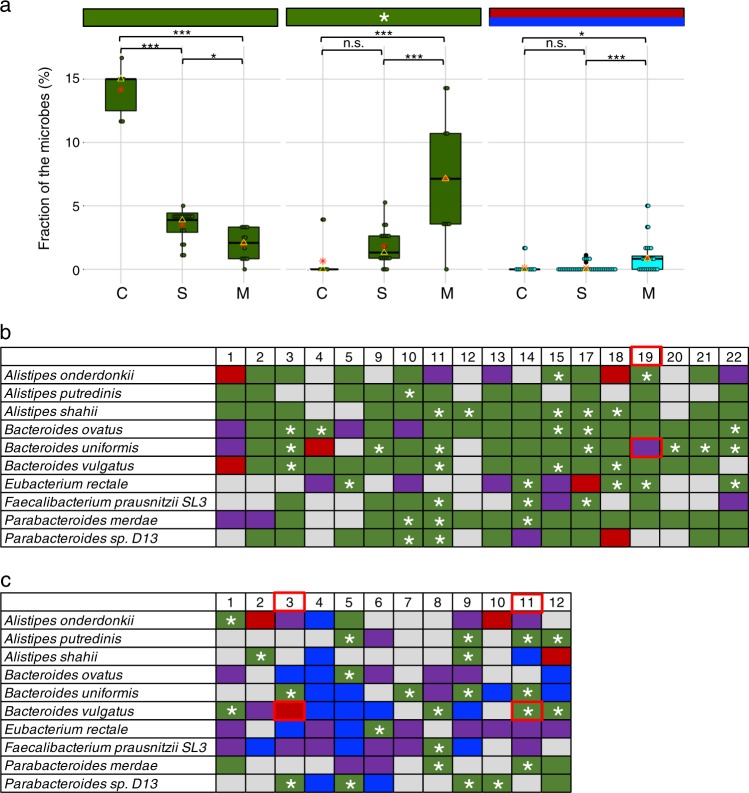
Summarized WSS scores. The top 10 species that were abundant across all individuals (*n* = 36) from the three data sets (control and single antibiotic data sets from Raymond et al.,^8^ and multiple antibiotics data set from Palleja et al.^9^) were selected to compare the WSS scores between every possible pair of samples per each individual. **a** The boxplots show the fraction of the top 10 species of each data set (C = control, S = single antibiotic, and M = multiple antibiotics) that fall into the respective color box group indicated by the horizontal color-coded bars (colors described in the main text). Values from the red and blue color box groups were merged to represent a single boxplot per each data set. The boxplots display a median (a yellow triangle), a mean (a red asterisk), interquartile range boxes. Each dot in the boxplot represents a value observed per individual in each data set, and the whiskers of the box are extended to the lowest and highest value observed in each data set. Significant differences (*P* value <0.05) between each data set were tested using an ANOVA followed by Tukey’s multiple-comparisons post hoc tests in R (version 3.5.1), and represented as a black asterisk above the boxplot; *P value <0.05, **P value <0.01, ***P value <0.001, n.s. = not significant (see Supplementary Data 1 for detailed values). **b**, **c** The summarized WSS scores of the top 10 species per individual from **b**, single antibiotic, and **c** multiple antibiotics data set were grouped into different color boxes (colors described in the main text). Each column in the table represents an individual and matches to the number shown in the Supplementary Data 1. WSS scores for all identified species are provided in Supplementary Data 1, and the summarized WSS scores for the control data set shown in the Supplementary Fig. 4. Additional strain profiling analysis was conducted for *B. uniformis* from individual #19 from Raymond et al. and for *B. vulgatus* from individual #3 and #11 from Palleja et al. (red outlined boxes; result from this analysis shown in Fig. 2)

A WSS analysis of each species in these two studies against the cut-off value showed the relatedness of the sample pairs at various time points (Fig. 1, Supplementary Figs. 1–3). The subsequent WSS relationships between each species were based on the cut-off values previously established by Kumar et al.^7^ to distinguish a related strain pair (WSS score > cut-off) from a non-related strain pair (WSS score < cut-off). The patterns of relatedness were categorized into color-coded groups in Fig. 1a–c as follows. The green boxes represent strain pairs from the same species that were related throughout the entire time points (always with the pre-strain). The green boxes with a white asterisk (*) represent a subset of these results where the strain pairs were only related between pre and last day post-treatment samples, whereas the intermediate time points had unrelated strain pairs (appearance of the transient strain was eventually replaced by the pre-strain). The red boxes represent the strain pairs were only related when the Day 7 (Raymond et al.^8^) or Day 8 (Palleja et al.^9^) samples were compared to the remaining post-treatment samples. The blue boxes represent the strain pairs were related when the Day 42 (Palleja et al.^9^) samples were compared to the last day of post-treatment samples. The purple boxes represent strain pairs belonging to the indicated species that showed no relationship between pre- and postantibiotic(s) strains. The gray boxes indicate microbial strains that we were unable to reliably determine relatedness due to the majority of the sample pairs did not satisfy the criteria of WSS analysis (minimum coverage of two genomes > 30% after filtering low coverage windows), or abundances of the species was low/absent for the majority of the time points (taxonomic composition data was reported in Palleja et al.^9^ and Raymond et al.^8^).

The top 10 most abundant species found across all individuals included members of the genera *Alistipes*, *Bacteroides*, *Eubacterium*, *Faecalibacterium*, and *Parabacteroides* (Supplementary Data 1). In the control data set, most of the individual pairs showed the original strain on the last day of the post-treatment period (Fig. 1a, Supplementary Fig. 4), a result similar to what we have previously found for the HMP data set.^10^ The control data set showed a significantly higher the fraction of original strains on the last day of post-treatment as compared to the single and multiple antibiotics data sets (Fig. 1a, Supplementary Data 1). However, the multiple antibiotics data set showed a significant increase of new strains as compared to the single antibiotic and the control data sets (Fig. 1a, Supplementary Data 1).

The WSS scores for the individual pairs at the intermediate times during the studies was next determined at Day 7 (Raymond et al.) and Day 8 and Day 42 (Palleja et al.) (Fig. 1b, c). Further analysis of the longitudinal samples revealed that in numerous individuals, transient strains were replaced with the pre-treatment strains (green with a white asterisk in Fig. 1b, c). The fraction of the transient strains was significantly higher in the multiple antibiotics data set as compared to the single antibiotic and the control data sets (Fig. 1a, Supplementary Data 1).

**Fig. 2:**
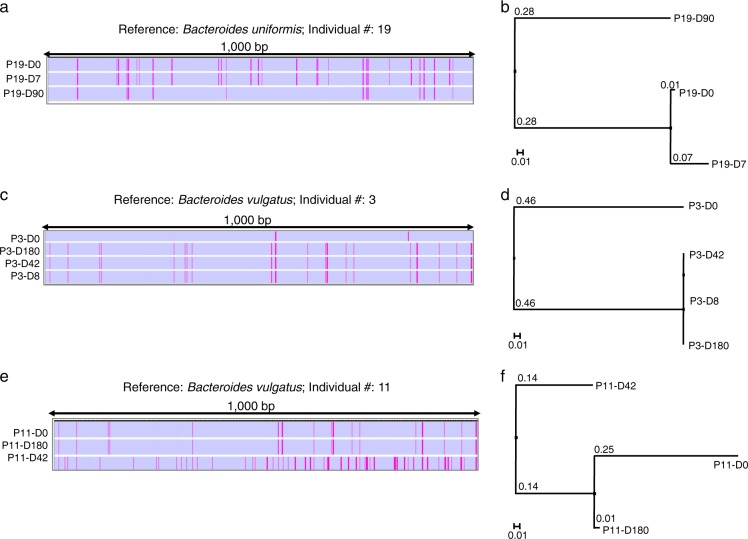
Strain profiling using Integrative Genomics Viewer and StrainPhlAn. The SNV patterns of the microbial genomic variants shown through Integrative Genomics Viewer (IGV) were randomly selected from a high-density SNV region (1000 base pairs length). **a** Individual #19 from Raymond et al. was selected to show the SNV patterns of the genomic variant against the reference sequence of *B. uniformis* at each time point. **c** Individual #3 and **e** Individual #11 were selected from Palleja et al. to show the SNV patterns of their genomic variants against the reference sequence of *B. vulgatus* at each time point. **b**, **d**, **f** For each selected individual, StrainPhlAn analysis was conducted. DNA sequences from species-specific marker genes were aligned and used to construct a neighbor-joining tree based on percentage identity (PID) distance between the marker genes through Jalview. The numbers at joining nodes indicate a PID. The tree is drawn to scale bar unit (0.1) displayed below the tree

To independently substantiate the identification of microbial strains, we used Integrative Genomics Viewer (IGV)^11^ and analysis by StrainPhlAn.^6^ IGV was used to visualize the overall genome-wide SNV identified from the WSS analysis of individual #19 from Raymond et al. In this example, WSS analysis on the *Bacteroides uniformis* reference sequences showed all WSS scores were below the cut-off compared to Day 0 vs. Day 90, and Day 7 vs. Day 90, indicating that the strain found at the last day of post-treatment was not related to either the pre- or post-antibiotic(s) strain. The SNV patterns depicted by IGV highlight the similarities between the pre and Day 7 and the differences with the SNV at Day 90 (Fig. 2a). Correspondingly, a distinct strain was identified at Day 90 as evidenced by the different clustering pattern as determined by StrainPhlAn (Fig. 2b). A similar situation was found in individual #19, where a new strain seen after antibiotics had replaced the pre-antibiotic strain (additional examples of this type of dynamic are shown in Supplementary Fig. 5). In Palleja et al., the new strain of *Bacteroides vulgatus* from individual #3 was not related with the original SNV pattern (Fig. 2c). StrainPhlAn analysis confirmed the pre-antibiotic *B. vulgatus* was clustered separately from the new strain present after antibiotics (Fig. 2d).

We noted several instances where there were blooms of new strains (different from the pre-strain) that were transient and eventually replaced by the pre-strain (green with a white asterisk in Fig. 1). Most probably, the initial bloom of the new strain is due to the more effective competition for nutrients as compared to the pre-antibiotic strain although it could also be a greater susceptibility of the pre-antibiotic strain to the antibiotics.^12–14^ As the overall microbial community structure recovers, the pre-antibiotic strain regains the competitive edge.^14,15^ The *B. vulgatus* from individual #11 from the Palleja et al. data was selected as an example of this situation (Fig. 2e). The IGV depiction of the pre-antibiotic and Day 180 strains are clearly different from the strain found at Day 42. However, this strain was completely replaced by Day 180 with a strain that was related, as determined from the WSS, to the pre-antibiotic strain. In support of this, StrainPhlAn analysis showed a distinct clade for the Day 42 strain (Fig. 2f). Thus, in this case, the new strain that appeared after the antibiotics was transient and eventually replaced by the pre-antibiotic strain.

**Fig. 3:**
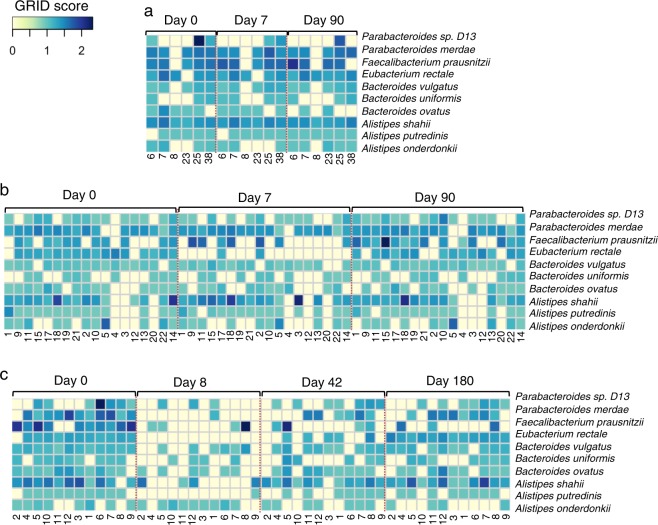
Microbe replication. Heatmap representing the Growth Rate InDex (GRiD)^16^ scores for the top 10 species that were abundant across all individuals (*n* = 36) determined at each time point for all individuals from the three data sets; **a** control and **b** single antibiotic data sets from Raymond et al., and **c** multiple antibiotics data set from Palleja et al. The heatmap was generated using the heatmap.2 function in R (version 3.5.1). Each number shown below the heatmap corresponds to the individual listed in Supplementary Data 1. The larger GRiD scores indicate a higher growth rate represented in dark blue, and the smaller GRiD scores represent a lower growth rate shown in light yellow (values <1.5 generally slow-growing microbes^16^). GRiD scores for all identified bacterial genomes as well as common species across all data sets were elaborated in Supplementary Data 1 and Supplementary Fig. 6, respectively. Significant differences (*P* value < 0.05) of the GRiD scores of the top 10 species between different time points, particularly Day 0 vs. the last day of the post-treatment sample in each data set were tested using an ANOVA followed by Tukey’s multiple-comparisons *post hoc* tests in R (version 3.5.1), showing no significant differences (*P* value >0.05) between Day 0 and Day 90 in both the control and single antibiotic data sets (see detailed values in Supplementary Data 1). Similarly, the multiple antibiotics data set showed no significant differences (*P* value >0.05) between Day 0 and Day 180 for the majority of species (9 out of 10 species; see detailed values in Supplementary Data 1)

One explanation for the replacement of strains post antibiotics would be differences in replication pre- and post-antibiotics. To address this issue, we characterized the microbial strains post antibiotics using a recently described informatics program to estimate the Growth Rate Index (GRiD) of the microbes.^16^ The GRiD score was calculated based on the coverage ratio of the peak (origin of replication, *ori*) and trough (terminus, *ter*) regions and found to be directly proportional to the growth rate of the microbe.^16^ A total of 209 bacterial genomes, the members of *Alistipes*, *Bacteroides*, Clostridiales, *Escherichia*, *Eubacterium*, *Faecalibacterium*, *Klebsiella*, and *Parabacteroides* were identified using this program as common species, showing different GRiD scores at each of the time points per individual from the two data sets (Supplementary Data 1, Supplementary Fig. 6). To be consistent with WSS analyses, the same top 10 species were selected from 209 bacterial genomes to show GRiD scores at each of the time points per individual from the two data sets (Fig. 3). In the control and single antibiotic data sets, the GRiD scores of the top 10 species showed no significant differences over time (Fig. 3a, b, Supplementary Data 1). The GRiD scores of the multiple antibiotics data set showed that the majority of the top 10 species were noticeably reduced from Day 0 to Day 8 in most individuals following the antibiotics (Fig. 3c). Most importantly, the GRiD scores for the 9 out of 10 species (with exception of *Faecalibacterium prausnitzii*) showed no significant differences between pre- and post-antibiotic(s) treatment samples, suggesting that the presence of these new strains was not due to enhanced replication compared to the pre-antibiotic strains (Fig. 3c, Supplementary Data 1). Palleja et al. noted a significant depletion of most of *F. prausnitzii* by Day 180.^9^ Examination of the individual GRiD scores revealed 9 out of the 12 participants’ GRiD scores of *F. prausnitzii* at Day 180 were absent, thus skewing the statistical analysis difference between pre- and post-antibiotic(s) treatment samples (Supplementary Data 1). Thus, the replacement of post antibiotic strains cannot be explained by differences in replication for the new strains.

It is possible that these strain changes could reflect the microbial adaptation to the new post antibiotic microbial community.^17^ Consistent with this possibility, a recent study characterized a model that showed for the recovery of the Palleja et al. microbial communities from antibiotics that there was a transition to a “new alternative stable state”.^18^ Since young healthy adults were used in this study, they would be expected to have stable microbial community functions.^2,19^ As shown from our analysis though, the capacity to recover with respect to the number and stability of new strains is specific for each individual. It is possible that as the individual ages, with each having differences in numbers and cycles of antibiotic treatment, the reservoir of microbial strains are depleted resulting in an intra-individual recovery pattern for specific microbial strains. Understanding this recovery pattern including the occurrence of particular strains following antibiotics may be an important consideration for long-term health, as it might impact strain-mediated colonization resistance or mechanisms of active antagonism.^20^ In the future, the characterization of these individual specific recovery patterns could also potentially be used to forecast the susceptibility to both endogenous and exogenous microbe pathogens.

## Methods

### Total sequence reads and processing

A total of 6,578,856,142 metagenomics sequencing reads were downloaded from two data sets: 4,864,146,612 reads from the Raymond et al. and 171,470,953 from the Palleja et al. (Supplementary Data 1). For intra-individual comparison, all of the individual’s samples from the two data sets were randomly subsampled (seed = 1000) to an average value of 35 million reads with seqtk (version 1.3) (https://github.com/lh3/seqtk). Subsampling of sequence reads showed no significant differences in the WSS scores in the respective samples of the two data sets. Sequence reads were filtered to remove adapters, low-quality reads (sliding window of 50 bases having a Q score <20), and short sequences (sequence length <50 bases) using Trimmomatic (version 0.36).^21^ After quality-based trimming and filtering processes, a total 3,883,083,696 sequences were used for the downstream analyses (Supplementary Data 1).

### Strain profiling using WSS analysis and StrainPhlAn

For the Window-based single-nucleotide variant (SNV) similarity (WSS) analysis, high-quality subsampled reads were aligned to the 93 reference sequences, which were previously constructed based on the Human Microbiome Project (HMP) data set^7,22^ using the Burrows-Wheeler aligner program (BWA; version 0.7.13).^23^ Multi-sample SNVs for each given reference sequence were then measured among all samples for each individual. To do this, SNVs were called for each sample using Genome Analysis Toolkit (GATK; version 3.7).^24^ The resultant Variant Call Format (VCF) files representing each achievable microbial genome variant from the 93 reference sequences were then used for pairwise comparisons between every possible pair of samples to measure their overall genome-wide SNV similarity. Any genome variant with a low sequence coverage (<30%) against their given reference sequences were excluded from the pairwise comparisons between samples. Low coverage windows with more than 50% of the bases having a read depth <5 were ignored to compare SNV similarity between sample pair. Also, the SNV loci having heterogeneity of >20% were excluded.^7^ A total of 30 and 37 species were found from the Raymond et al. and the Palleja et al. data sets, respectively, after the filtering process (Supplementary Data 1). The top 10 species were then selected based on the obtainability of a WSS score averaged across all samples, specifically when the pre-treatment samples were compared with the last day of the post-treatment samples (i.e. Day 90 for Raymond et al. and Day 180 for Palleja et al.). The resultant WSS scores including the previously determined pairwise similarity cut-off value for each species^7,10^ were then visualized across all individuals using the ggplot2 package (version 3.1.1) (https://cran.r-project.org/web/packages/ggplot2/index.html) in R (version 3.5.1) software.^25^ To determine the relatedness of SNV similarity at different time points for each individual, WSS scores from all pairwise comparisons were compared against each other, summarized, and visualized using Microsoft Excel (Microsoft, Seattle, WA, USA).

In order to visualize the SNV patterns at different time points for each individual, the VCF file generated for the specific strains of each sample were uploaded to Integrative Genomics Viewer (IGV; version 2.4.10)^11^ and aligned to their reference sequences. In particular, two individuals (#3 and #11) from the Palleja et al. data and one individual (#19) from the Raymond et al. data were selected to show their SNV patterns at each time point. *B. vulgatus* and *B. uniformis* were used as reference sequences for the selected individuals. A random high-density SNV region (1000 bp length) was selected for visualization among the entire sequence of *B. vulgatus* and *B. uniformis* using IGV.^11^

Strain profiling was also conducted for the selected individuals (#3 and #11 from Palleja et al. data and #19 from Raymond et al. data) using StrainPhlAn.^6^ To do this, the high-quality subsampled reads were mapped against the set of species-specific marker gene database established in MetaPhlAn.^26^ The sample-specific markers were reconstructed by using the variant calling approach, and then the reconstructed markers were used to build a phylogeny of the strains.^6^ The phylogenetic tree was visualized using the neighbor-joining method in Jalview.^27^

### Growth dynamics of microbes using GRiD-MG

We have applied the Growth Rate InDex—MetaGenomic (GRiD-MG) approach to the two data sets to estimate in situ growth rates of microbes in a community.^16^ The high-quality metagenomics reads from the two data sets were mapped against the GRiD-MG database, which included 32,819 representative bacterial genomes.^16^ The mapped reads were then re-assigned using Pathoscope (version 2.0)^28^ with default parameters. Any genomes with a coverage value below 0.2× and a high species heterogeneity (>0.3) were discarded. After the filtering process, a total 209 species were commonly found across all the data sets. To be consistent with the WSS analysis, we used the top 10 species to show the GRiD scores per each sample for each individual. The resultant GRiD scores were summarized across all samples and then used to generate the heatmap using “heatmap.2” function (http://CRAN.R-project.org/package=gplots) in R software (version 3.5.1).^25^

### Statistical analysis

Statistical significance (*P* value <0.05) was determined by using one-way ANOVA followed by Tukey’s multiple-comparisons post hoc test in R software (version 3.5.1),^25^ as appropriate and indicated in the figure legends and the main text. Detailed values were shown in Supplementary Data 1. Boxplots were generated using the ggplot2 package (version 3.1.1) in R (version 3.5.1).

### Reporting summary

Further information on research design is available in the Nature Research Reporting Summary linked to this article.

## Supplementary information

Supplementary Information

Supplementary Data 1

Reporting Summary

## Data Availability

The original sequencing data set of the stool samples used in this study were downloaded from the European Nucleotide Archive (accession numbers: PRJEB8094 for Raymond et al.^8^ and ERP022986 for Palleja et al.^9^).
