# Supplementary material for: Individualized recovery of gut microbial strains post antibiotics
Source: NPJ Biofilms Microbiomes. 2019 Oct 11;5:30. doi: 10.1038/s41522-019-0103-8 (PMC6789009; doi:10.1038/s41522-019-0103-8)
Supplement: Supplementary file 1 — Supplementary Information [file 41522_2019_103_MOESM1_ESM.pdf]

# Individualized Recovery of Gut Microbial Strains Post Antibiotics

Hyunmin Koo[1*], Joseph A. Hakim[2], David K. Crossman[1], Ranjit Kumar[3], Elliot J. Lefkowitz[4], and Casey D. Morrow[5*]

[1]Department of Genetics and Heflin Center for Genomic Science

University of Alabama at Birmingham

Birmingham, Alabama 35294

[2]Department of Biology

University of Alabama at Birmingham

Birmingham, Alabama 35294

[3]Biomedical Informatics

Center for Clinical and Translational Sciences

University of Alabama at Birmingham

Birmingham, Alabama 35294

[4]Department of Microbiology

University of Alabama at Birmingham

Birmingham, Alabama 35294

[5]Department of Cell, Developmental and Integrative Biology

University of Alabama at Birmingham

Birmingham, Alabama 35294

## Description of Supplementary Information:

Supplementary Information file includes Supplementary Figures 1-6.

## Supplementary Figures

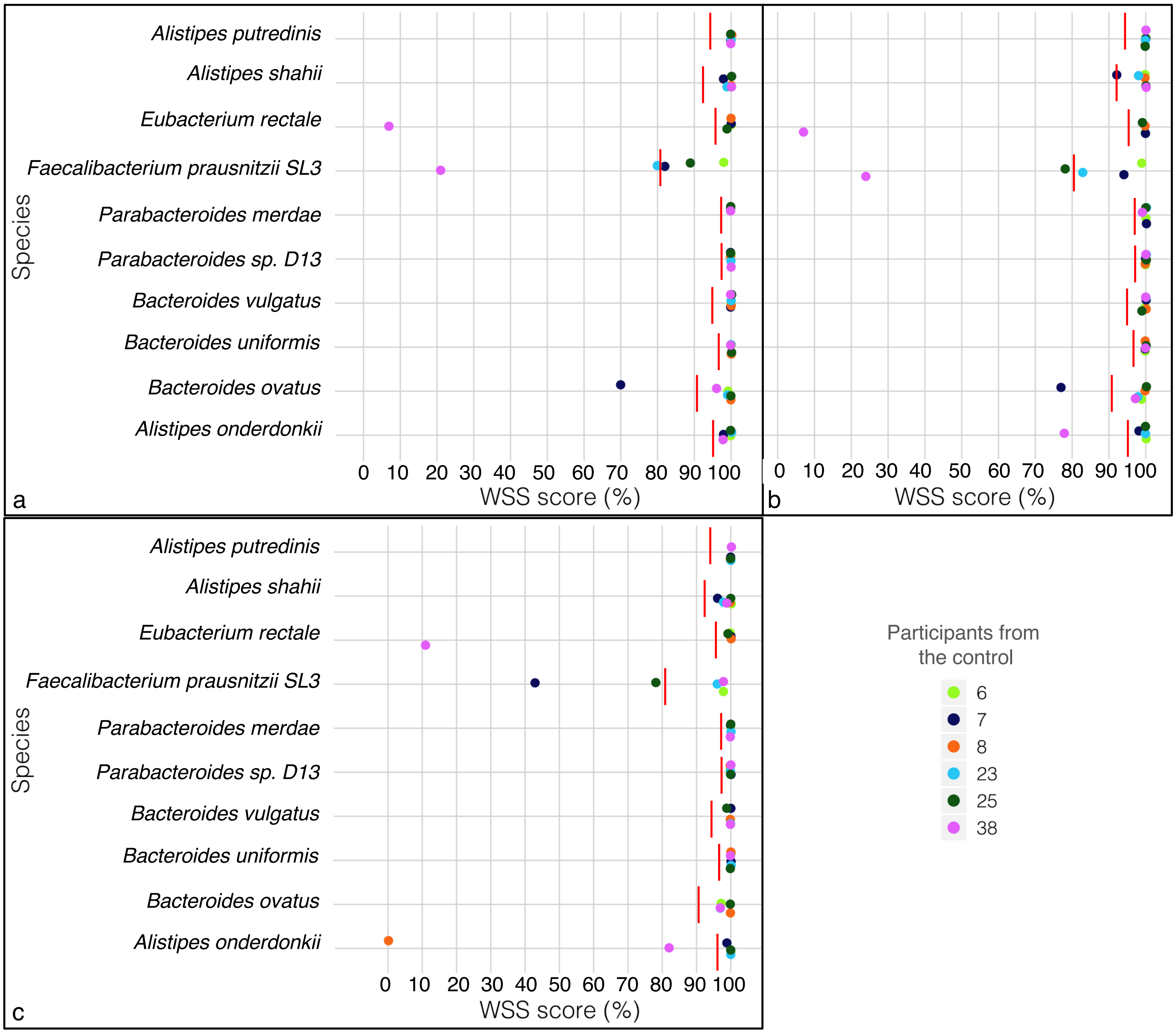
Alistipes putredinis
Alistipes shahii
Eubacterium rectale
Faecalibacterium prausnitzii SL3
Parabacteroides merdae
Parabacteroides sp. D13
Bacteroides vulgatus
Bacteroides uniformis
Bacteroides ovatus
Alistipes onderdonkii
Species
0 10 20 30 40 50 60 70 80 90 100
WSS score (%)
a
b
c
Participants from the control
6
7
8
23
25
38

**Supplementary Figure 1: WSS analysis for the control data set from Raymond et al.** The resultant WSS scores of 6 participants including the cut-off value (red line) were represented in a scatter plot through ggplot2 package in R software. Pairwise comparisons were conducted between **a**, Day 0 vs. Day 7, **b**, Day 0 vs. Day 90, and **c**, Day 7 vs. Day 90 across six participants to measure overall genome-wide SNV similarity.

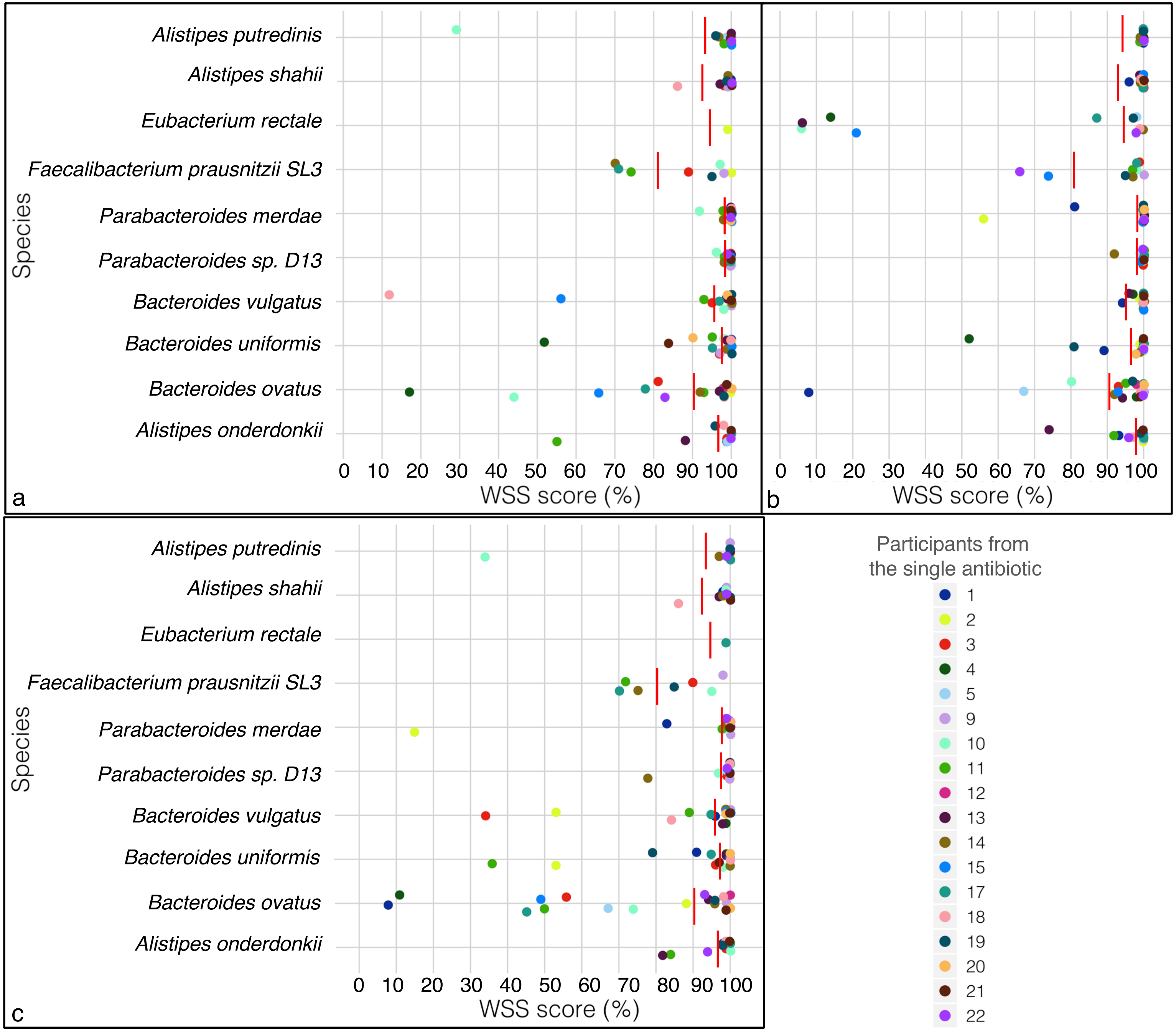

Alistipes putredinis
Alistipes shahii
Eubacterium rectale
Faecalibacterium prausnitzii SL3
Parabacteroides merdae
Parabacteroides sp. D13
Bacteroides vulgatus
Bacteroides uniformis
Bacteroides ovatus
Alistipes onderdonkii
Species
0 10 20 30 40 50 60 70 80 90 100
WSS score (%)
a
b
c
Participants from the single antibiotic
1
2
3
4
5
9
10
11
12
13
14
15
17
18
19
20
21
22

**Supplementary Figure 2: WSS analysis for the single antibiotic data set from Raymond et al.** WSS scores of 18 participants including the cut-off value (red line) were displayed in a scatter plot through ggplot2 package in R software. Pairwise comparisons were performed between **a**, Day 0 vs. Day 7, **b**, Day 0 vs. Day 90, and **c**, Day 7 vs. Day 90 across 18 participants to measure overall genome-wide SNV similarity.

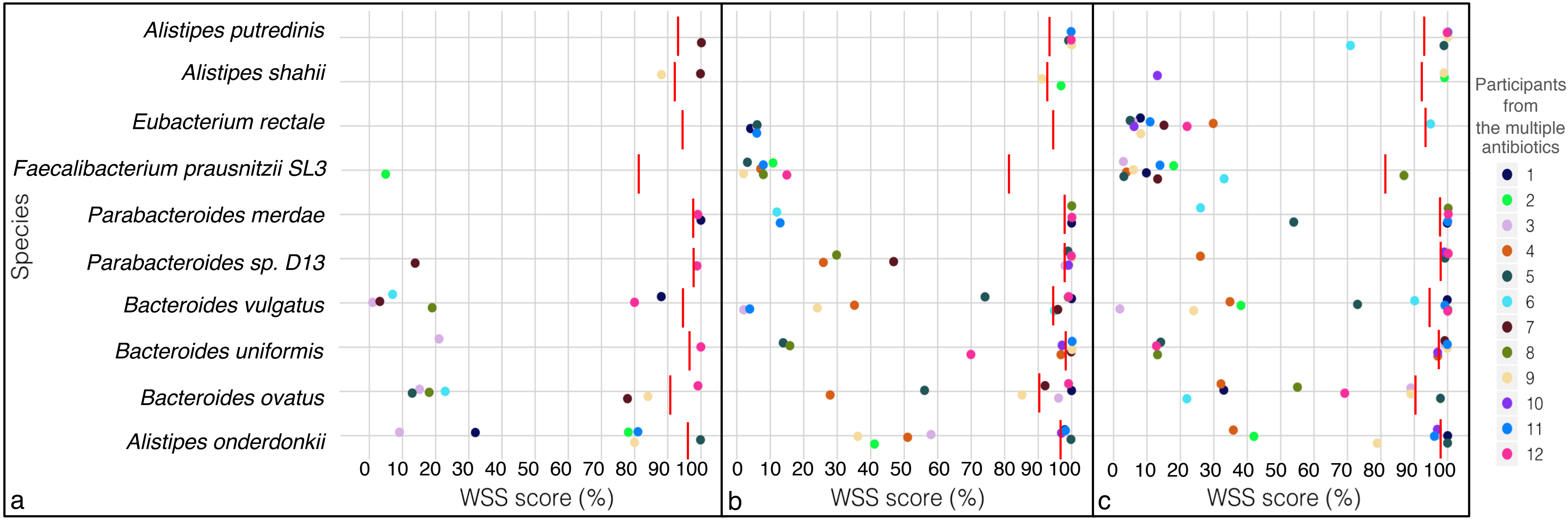
Alistipes putredinis
Alistipes shahii
Eubacterium rectale
Faecalibacterium prausnitzii SL3
Parabacteroides merdae
Parabacteroides sp. D13
Bacteroides vulgatus
Bacteroides uniformis
Bacteroides ovatus
Alistipes onderdonkii
Species
0 10 20 30 40 50 60 70 80 90 100
WSS score (%)
a
b
c
Participants from the multiple antibiotics
1
2
3
4
5
6
7
8
9
10
11
12

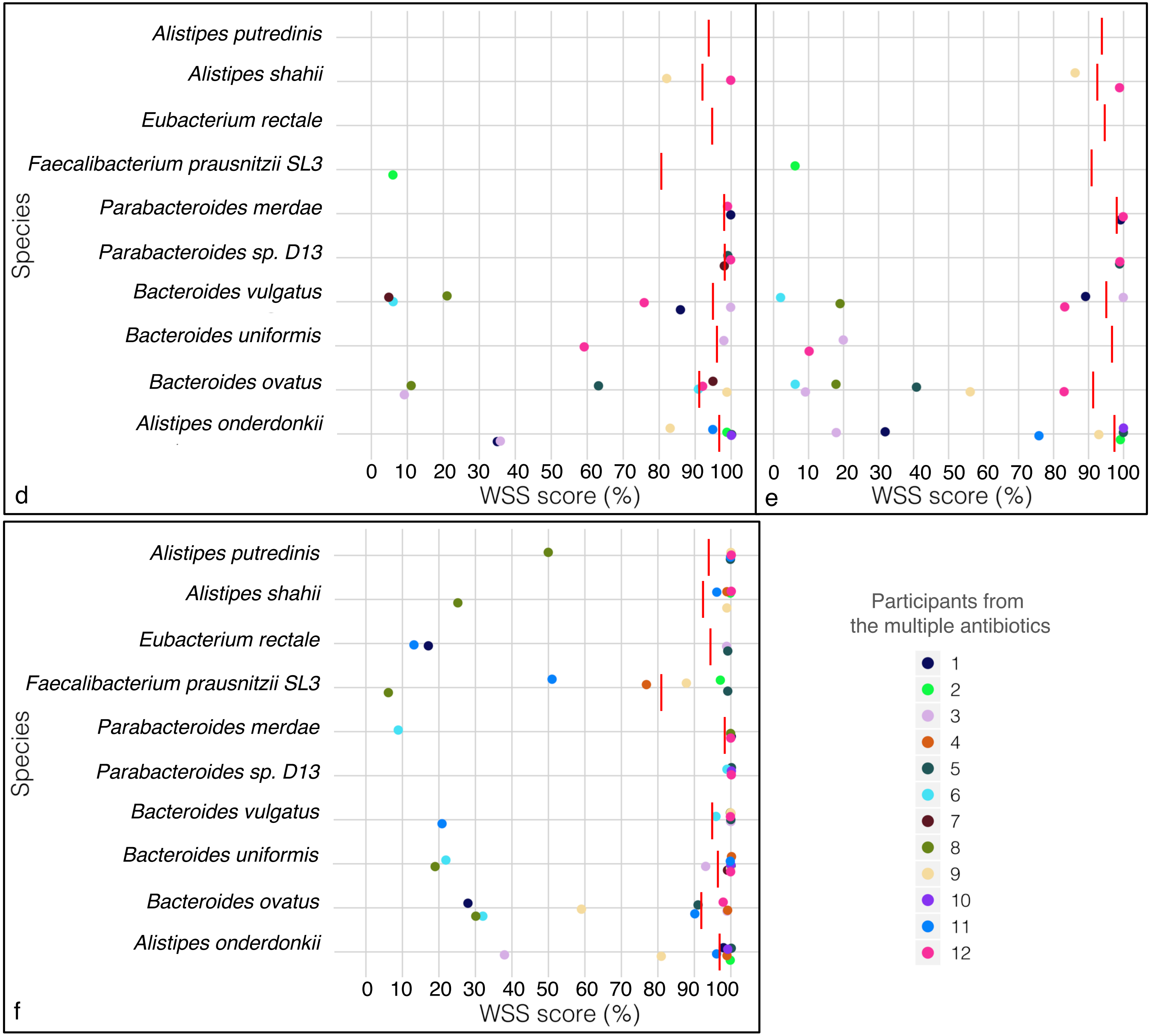
Alistipes putredinis
Alistipes shahii
Eubacterium rectale
Faecalibacterium prausnitzii SL3
Parabacteroides merdae
Parabacteroides sp. D13
Bacteroides vulgatus
Bacteroides uniformis
Bacteroides ovatus
Alistipes onderdonkii
Species
0 10 20 30 40 50 60 70 80 90 100
WSS score (%)
d
e
f
Participants from
the multiple antibiotics
1
2
3
4
5
6
7
8
9
10
11
12

**Supplementary Figure 3: WSS analysis for the multiple antibiotics data set from Palleja et al.** WSS scores of 12 participants including the cut-off value (red line) were shown in a scatter plot through ggplot2 package in R software. Pairwise comparisons were performed between **a**, Day 0 vs. Day 8, **b**, Day 0 vs. Day 42, **c**, Day 0 vs. Day 180, **d**, Day 8 vs. Day 42, **e**, Day 8 vs. Day 180, and **f**, Day 42 vs. Day 180 across 12 participants to measure overall genome-wide SNV similarity.

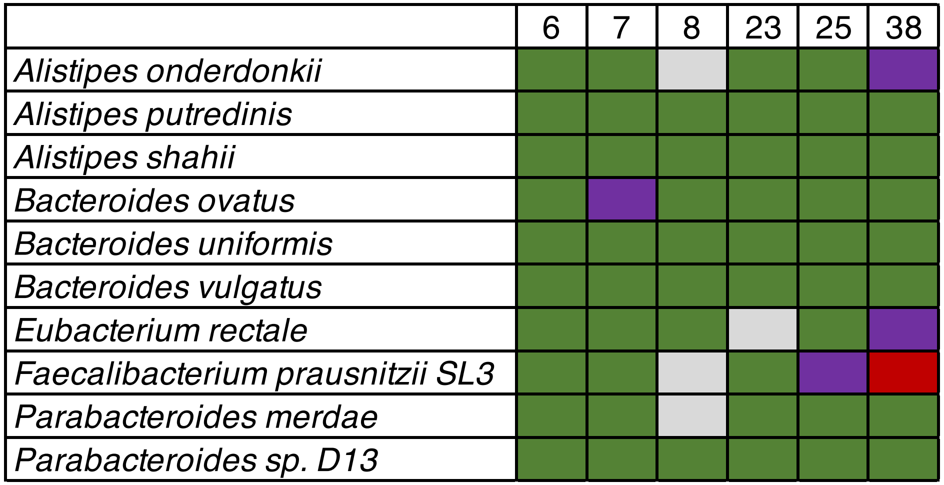


**Supplementary Figure 4**: **Summarized WSS scores for the control data set from Raymond et al.** The top 10 species that were abundant across all individuals (*n*=36) from the three data sets (control and single antibiotic data sets from Raymond et al., and multiple antibiotics data set from Palleja et al.) were selected to compare the WSS scores between every possible pair of samples per each individual. The subsequent WSS relationships between each species were based on the cut-off values previously established by Kumar et al. to distinguish a related strain pair (WSS score > cut-off) from a non-related strain pair (WSS score < cut-off). The patterns of relatedness were categorized into color-coded groups as follows. The green boxes represent strain pairs from the same species that were related throughout the entire time points (always the pre-strain). The red boxes represent the strain pairs were only related when the Day 7 samples were compared to the remaining post-treatment samples. The purple boxes represent strain pairs belonging to the indicated species that showed no relationship between pre- and post-antibiotic strains. The gray boxes indicate that ignored WSS scores due to low genome coverage (< 30%) for the sample pair after after filtering low coverage windows.

a

| **Day0 vs. Day7** | 1 | 2 | 3 | 4 | 5 | 9 | 10 | 11 | 12 | 13 | 14 | 15 | 17 | 18 | 19 | 20 | 21 | 22 |
|---|---|---|---|---|---|---|---|---|---|---|---|---|---|---|---|---|---|---|
| *Alistipes onderdonkii* | | | | NS | | NS | | 55 | NS | 88 | NS | NS | | | | NS | | |
| *Alistipes putredinis* | | | NS | NS | | | | | NS | | | | | NS | | NS | | |
| *Alistipes shahii* | | | | NS | NS | | | NS | NS | | | NS | NS | | | NS | | |
| *Bacteroides ovatus* | | | | | | | 44 | | | | | | | | | | | |
| *Bacteroides uniformis* | | | | | NS | | | | NS | | | | | | | | | NS |
| *Bacteroides vulgatus* | | | | | | | | | NS | | | | | | | | | NS |
| *Eubacterium rectale* | NS | NS | NS | NS | NS | NS | NS | NS | NS | NS | NS | NS | NS | NS | NS | NS | NS | NS |
| *Faecalibacterium prausnitzii SL3* | NS | NS | | NS | NS | | | | NS | NS | | NS | | NS | | NS | NS | NS |
| *Parabacteroides merdae* | | 98 | | NS | NS | | | | | | | | | | | | | |
| *Parabacteroides sp. D13* | | | | NS | | | | | NS | | 98 | | | NS | NS | NS | | |

| **Day0 vs. Day90** | 1 | 2 | 3 | 4 | 5 | 9 | 10 | 11 | 12 | 13 | 14 | 15 | 17 | 18 | 19 | 20 | 21 | 22 |
|---|---|---|---|---|---|---|---|---|---|---|---|---|---|---|---|---|---|---|
| *Alistipes onderdonkii* | | | | NS | | NS | | 92 | NS | 74 | NS | NS | | | | NS | | 96 |
| *Alistipes putredinis* | | | NS | NS | | | | | NS | | | NS | | NS | | NS | NS | |
| *Alistipes shahii* | | | | NS | NS | | | | | | | | | | | NS | | |
| *Bacteroides ovatus* | 8 | | | | 67 | | 80 | | | | | | | | | | | |
| *Bacteroides uniformis* | 89 | | | | NS | | | | NS | | | | | | 81 | | | |
| *Bacteroides vulgatus* | | | | | | | | | NS | | | | | | | | | NS |
| *Eubacterium rectale* | NS | NS | | 14 | | | 6 | NS | NS | 6 | | 21 | | | | NS | NS | |
| *Faecalibacterium prausnitzii SL3* | NS | NS | | NS | NS | | | | NS | NS | | 74 | | NS | | NS | NS | 66 |
| *Parabacteroides merdae* | 81 | 56 | | NS | NS | | | | | | | | | | | | | |
| *Parabacteroides sp. D13* | NS | | | NS | | | | | NS | | 92 | | | NS | NS | NS | | |

WSS score all below the cut-off
NS No Score

| **Day7 vs. Day90** | 1 | 2 | 3 | 4 | 5 | 9 | 10 | 11 | 12 | 13 | 14 | 15 | 17 | 18 | 19 | 20 | 21 | 22 |
|---|---|---|---|---|---|---|---|---|---|---|---|---|---|---|---|---|---|---|
| *Alistipes onderdonkii* | | | | NS | | NS | | 84 | NS | 82 | NS | NS | | | | NS | | 94 |
| *Alistipes putredinis* | | | NS | NS | | | | | NS | | | NS | | NS | | NS | NS | |
| *Alistipes shahii* | | | | NS | NS | | | | | | | | | | | NS | | |
| *Bacteroides ovatus* | 8 | | | | 67 | | 74 | | | | | | | | | | | |
| *Bacteroides uniformis* | 91 | | | | NS | | | | NS | | | | | | 79 | | | |
| *Bacteroides vulgatus* | | | | | | | | | NS | | | | | | | | | NS |
| *Eubacterium rectale* | NS | NS | NS | NS | NS | NS | NS | NS | NS | NS | NS | NS | | NS | NS | NS | NS | NS |
| *Faecalibacterium prausnitzii SL3* | NS | NS | | NS | NS | | | | NS | NS | | NS | | NS | | NS | NS | NS |
| *Parabacteroides merdae* | 83 | 15 | | NS | NS | | | | | | | | | | | | | |
| *Parabacteroides sp. D13* | NS | | | NS | | | | | NS | | 78 | | | | NS | NS | | |

b

| Day0 vs. Day8 | 1 | 2 | 3 | 4 | 5 | 6 | 7 | 8 | 9 | 10 | 11 | 12 |
|---|---|---|---|---|---|---|---|---|---|---|---|---|
| *Alistipes onderdonkii* | | | 9 | | | | | | 80 | | 81 | |
| *Alistipes putredinis* | | | | | | NS | | | | | | |
| *Alistipes shahii* | | | | | | | | | | | | |
| *Bacteroides ovatus* | NS | | | | | 23 | | 18 | 84 | | | |
| *Bacteroides uniformis* | | | | | | | | 2 | | | | |
| *Bacteroides vulgatus* | | NS | | | | | | | | | | |
| *Eubacterium rectale* | NS | | | NS | | | NS | | NS | NS | NS | NS |
| *Faecalibacterium prausnitzii SL3* | NS | | NS | NS | NS | NS | NS | | | | NS | |
| *Parabacteroides merdae* | | | | | NS | NS | | | | | | |
| *Parabacteroides sp. D13* | | | | | | | | | | | | |

| Day0 vs. Day42 | 1 | 2 | 3 | 4 | 5 | 6 | 7 | 8 | 9 | 10 | 11 | 12 |
|---|---|---|---|---|---|---|---|---|---|---|---|---|
| *Alistipes onderdonkii* | | | 58 | | | | | | 36 | | 98 | |
| *Alistipes putredinis* | | | | | | NS | | | | | | |
| *Alistipes shahii* | | | | | | | | | | | | |
| *Bacteroides ovatus* | 100 | | | | | NS | | NS | 85 | | | |
| *Bacteroides uniformis* | | | | | | | | 16 | | | | |
| *Bacteroides vulgatus* | | NS | | | | | | | | | | |
| *Eubacterium rectale* | 4 | | | NS | | | NS | | NS | NS | 6 | NS |
| *Faecalibacterium prausnitzii SL3* | NS | | NS | 7 | 3 | NS | NS | | | | 8 | |
| *Parabacteroides merdae* | | | | | NS | 12 | | | | | | |
| *Parabacteroides sp. D13* | | | | | | | | | | | | |

| Day0 vs. Day180 | 1 | 2 | 3 | 4 | 5 | 6 | 7 | 8 | 9 | 10 | 11 | 12 |
|---|---|---|---|---|---|---|---|---|---|---|---|---|
| *Alistipes onderdonkii* | | | 97 | | | | | | 79 | | 96 | |
| *Alistipes putredinis* | | | | | | 71 | | | | | | |
| *Alistipes shahii* | | | | | | | | | | | | |
| *Bacteroides ovatus* | 33 | | | | | 22 | | 55 | 89 | | | |
| *Bacteroides uniformis* | | | | | | | | 13 | | | | |
| *Bacteroides vulgatus* | | 38 | | | | | | | | | | |
| *Eubacterium rectale* | 8 | | | 30 | | | 15 | | 8 | 6 | 11 | 22 |
| *Faecalibacterium prausnitzii SL3* | 10 | | 3 | 4 | 3 | 33 | 13 | | | | 14 | |
| *Parabacteroides merdae* | | | | | 54 | 26 | | | | | | |
| *Parabacteroides sp. D13* | | | | | | | | | | | | |

| | |
|---|---|
| (orange) | WSS score all below the cut-off |
| NS | No Score |

C

| Day8 vs. Day42 | 1 | 2 | 3 | 4 | 5 | 6 | 7 | 8 | 9 | 10 | 11 | 12 |
|---|---|---|---|---|---|---|---|---|---|---|---|---|
| *Alistipes onderdonkii* | | | 36 | | | | | | 83 | | 95 | |
| *Alistipes putredinis* | | | | | | NS | | | | | | |
| *Alistipes shahii* | | | | | | | | | | | | |
| *Bacteroides ovatus* | NS | | | | | 91 | | 11 | 99 | | | |
| *Bacteroides uniformis* | | | | | | | | NS | | | | |
| *Bacteroides vulgatus* | | NS | | | | | | | | | | |
| *Eubacterium rectale* | NS | | | NS | | | NS | | NS | NS | NS | NS |
| *Faecalibacterium prausnitzii SL3* | NS | | NS | NS | NS | NS | NS | | | | NS | |
| *Parabacteroides merdae* | | | | | NS | NS | | | | | | |
| *Parabacteroides sp. D13* | | | | | | | | | | | | |

| Day8 vs. Day180 | 1 | 2 | 3 | 4 | 5 | 6 | 7 | 8 | 9 | 10 | 11 | 12 |
|---|---|---|---|---|---|---|---|---|---|---|---|---|
| *Alistipes onderdonkii* | | | 32 | | | | | | 93 | | 76 | |
| *Alistipes putredinis* | | | | | | NS | | | | | | |
| *Alistipes shahii* | | | | | | | | | | | | |
| *Bacteroides ovatus* | NS | | | | | 6 | | 18 | 56 | | | |
| *Bacteroides uniformis* | | | | | | | | NS | | | | |
| *Bacteroides vulgatus* | | NS | | | | | | | | | | |
| *Eubacterium rectale* | NS | | | NS | | | NS | | NS | NS | NS | NS |
| *Faecalibacterium prausnitzii SL3* | NS | | NS | NS | NS | NS | NS | | | | NS | |
| *Parabacteroides merdae* | | | | | NS | NS | | | | | | |
| *Parabacteroides sp. D13* | | | | | | | | | | | | |

| Day42 vs. Day180 | 1 | 2 | 3 | 4 | 5 | 6 | 7 | 8 | 9 | 10 | 11 | 12 |
|---|---|---|---|---|---|---|---|---|---|---|---|---|
| *Alistipes onderdonkii* | | | 38 | | | | | | 81 | | 96 | |
| *Alistipes putredinis* | | | | | | NS | | | | | | |
| *Alistipes shahii* | | | | | | | | | | | | |
| *Bacteroides ovatus* | 28 | | | | | 32 | | 30 | 59 | | | |
| *Bacteroides uniformis* | | | | | | | | 19 | | | | |
| *Bacteroides vulgatus* | | NS | | | | | | | | | | |
| *Eubacterium rectale* | 17 | | | NS | | | NS | | NS | NS | 13 | NS |
| *Faecalibacterium prausnitzii SL3* | NS | | NS | 77 | 99 | NS | NS | | | | 51 | |
| *Parabacteroides merdae* | | | | | NS | 9 | | | | | | |
| *Parabacteroides sp. D13* | | | | | | | | | | | | |

Legend:
- (yellow) WSS score all below the cut-off
- NS No Score

**Supplementary Figure 5: WSS table for below the cut-off value.** Summarized tables represent WSS scores of **a**, Raymond et al., and **b**, and **c**, Palleja et al. below the cut-off value at each time points (shown in Figure 1 as purple shaded boxes). The gray shaded boxes with a “NS” represent no WSS scores due to low sequence coverage for one of the compared samples.

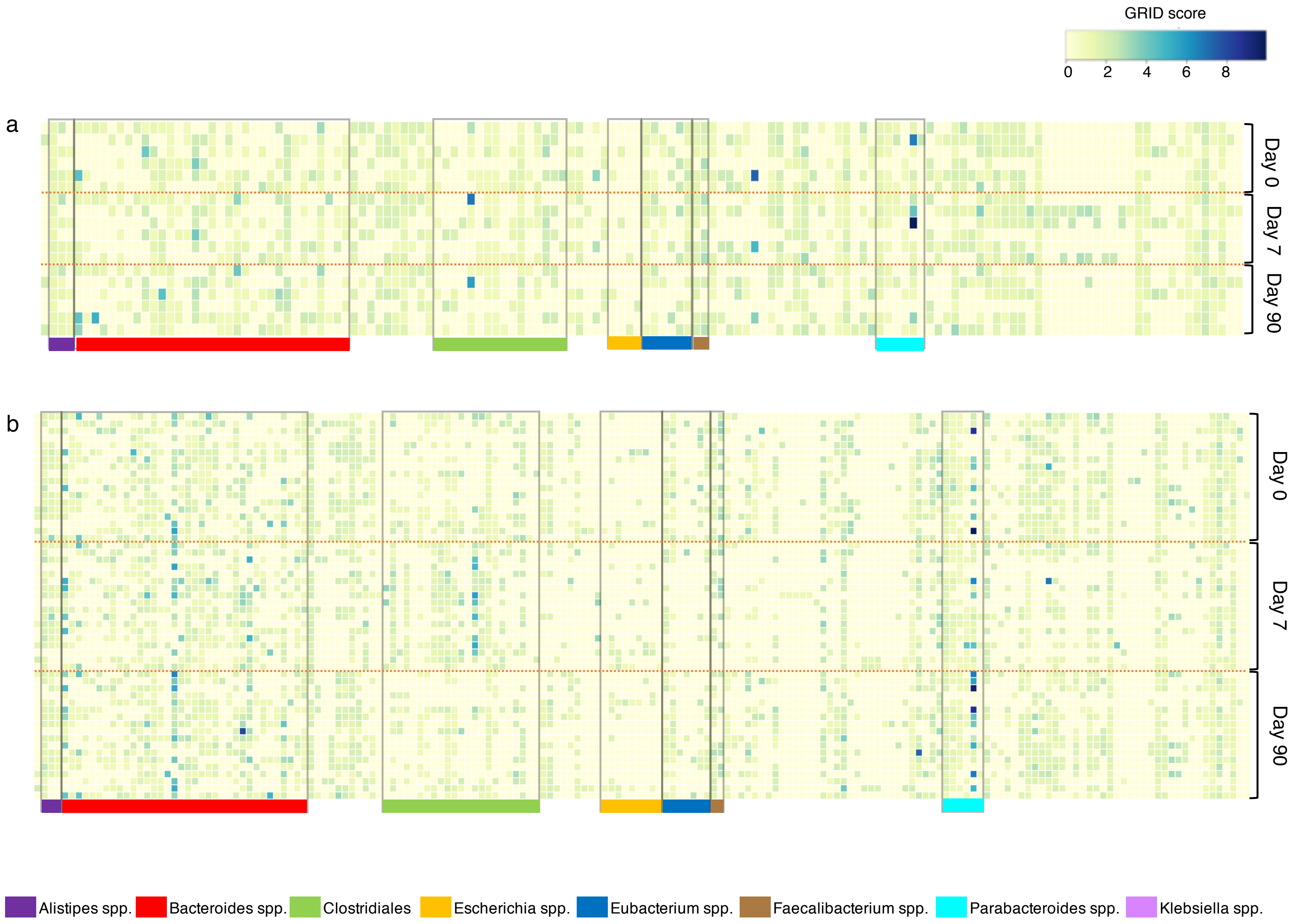
GRID score
0
2
4
6
8
a
b
Day 0
Day 7
Day 90
Alistipes spp.
Bacteroides spp.
Clostridiales
Escherichia spp.
Eubacterium spp.
Faecalibacterium spp.
Parabacteroides spp.
Klebsiella spp.

c

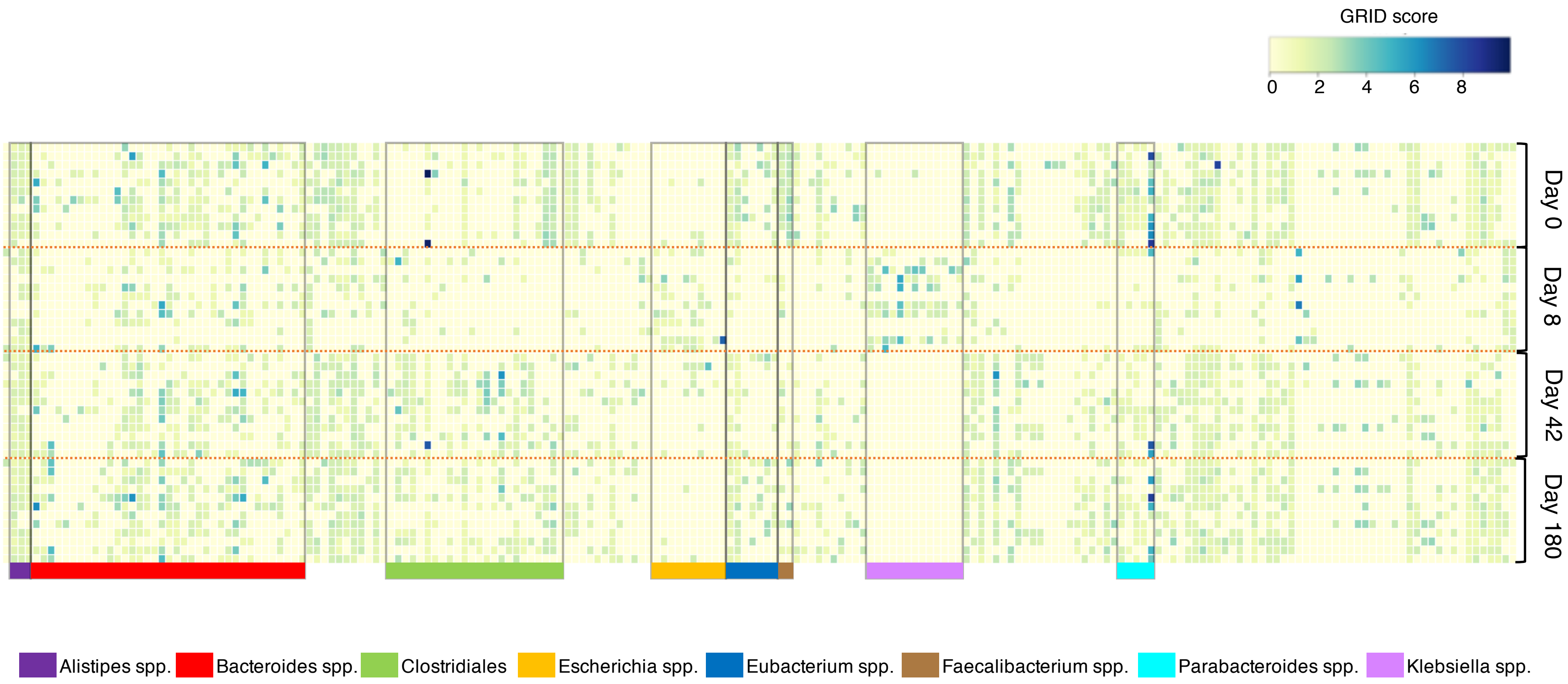


**Supplementary Figure 6**: **GRiD scores for 209 bacterial genomes.** Heatmap representing the Growth Rate InDex (GRiD) scores for the 209 bacterial genomes determined for all individuals at each time point. From the 209 bacterial genomes, members of order Clostridiales along with genus *Alistipes*, *Bacteroides*, *Escherichia*, *Eubacterium*, *Faecalibacterium*, *Parabacteroides*, and *Klebsiella* were emphasized to compare their GRiD scores over time. The colored side bars below the heatmap represent different groups of microbes. The larger GRiD scores indicate a higher growth rate represented in dark blue, and the smaller GRiD scores represent a lower growth rate shown in light yellow (values < 1.5 generally slow-growing microbes), GRiD scores for all identified bacterial genomes were elaborated in Supplementary Table 5. The plot was generated using the "heatmap.2" function in R software. **a**, control, **b,** single antibiotic, and **c,** multiple antibiotics data set.

## Description of Supplementary Data 1:

Supplementary Data 1 file includes Supplementary Tables 1-7 (Table S1-S7).

Name: Table S1
Description: Sequence reads information of each sample used in this study. The original sequence files were sequenced, preprocessed, and deposited by (A) Raymond et al. and (B) Palleja et al. To conduct the WSS analysis, all files were downloaded from the European Nucleotide Archive (accession numbers: PRJEB8094 and ERP022986). Each sample were then subsampled to 35 million reads. The table represents the sequence read count before and after quality checking, subsampling, and filtering processes.

Name: Table S2
Description: WSS results on the Raymond et al. study. All pairwise comparisons were conducted on the (A) Day 0 vs. Day 7 and 90; and the (B) Day 7 vs. Day 90 samples. The resultant WSS scores are shown as a numerical value along with the data bar graphs. The WSS scores which were above the cut-off (CO) values are in red.

Name: Table S3
Description: WSS results on Palleja et al. study. All pairwise comparisons were conducted on the (A) Day 0 vs. Day 8, 42, and 180; (B) Day 8 vs. Day 42 and 180; and (C) Day 42 vs. Day 180 samples. The resultant WSS scores are shown as a numerical value along with the data bar graphs. The WSS scores which were above the cut-off (CO) values are in red.

Name: Table S4
Description: The number of participants who were able to provide a WSS score (between Day 0 and Day 180 comparison) for each species.

Name: Table S5
Description: Significant differences (P-value < 0.05) of a fraction of the top 10 species from each data set (control and single antibiotic data sets from Raymond et al., and multiple antibiotics data set from Palleja et al.) that fall into the respective color box group (Green, Green with asterisk, and Red and Blue). Analyses were conducted using an ANOVA followed by Tukey's multiple-comparisons post hoc tests in R (version 3.5.1).

Name: Table S6(A)
Description: The results from the Growth Rate InDex - MetaGenomics (GRiD-MG) analysis on the control data set (n=6) from Raymond et al. The GRiD scores indicate the growth rate of each microbe represented in the community.

Name: Table S6(B)
Description: The results from the Growth Rate InDex - MetaGenomics (GRiD-MG) analysis on the single antibiotic data set (n=18) from Raymond et al. The GRiD scores indicate the growth rate of each microbe in the community.

Name: Table S6(C)
Description: The results from the Growth Rate InDex - MetaGenomics (GRiD-MG) analysis on the multiple antibiotics data set (n=12) from Palleja et al. The GRiD scores indicate the growth rate of each microbe in the community.

Name: Table S7
Description: Significant differences (P-value < 0.05) of the mean GRiD scores of the top 10 species between the Day 0 and last day post-treatment samples of each data set (control and single antibiotic data sets from Raymond et al. and multiple antibiotics data set from Palleja et al). For the multiple antibiotics data set, each species was selected to compare significant differences between Day 0 and Day 180 (last day of the post-treatment). In addition, the top 9 species (without Faecalibacterium prausnitzii) were selected to compare significant differences between the Day 0 and Day 180. All analyses were conducted using an ANOVA followed by Tukey's multiple-comparisons post hoc tests in R (version 3.5.1).
